# The First Macrolide-Resistant *Bordetella pertussis* Strains Isolated From Iranian Patients

**DOI:** 10.5812/jjm.10880

**Published:** 2014-06-01

**Authors:** Fereshteh Shahcheraghi, Masoumeh Nakhost Lotfi, Vajiheh Sadat Nikbin, Fahimeh Shooraj, Reza Azizian, Masoumeh Parzadeh, Mohammad Reza Allahyar Torkaman, Seyed Mohsen Zahraei

**Affiliations:** 1 Department of Bacteriology, Pertussis Reference Laboratory, Microbiology Research Center, Pasteur Institute of Iran, Tehran, IR Iran; 2 Center for Communicable Diseases Control, Ministry of Health and Medical Education, Tehran, IR Iran

**Keywords:** *Bordetella pertussis*, Macrolides, PFGE, Antibiotic Resistance

## Abstract

**Background:**

Whooping cough was considered as one of the major causes of childhood morbidity and mortality worldwide. Resistant isolates of *Bordetella pertussis* to macrolides in some countries have been recently reported.

**Objectives:**

Recent reports on macrolide-resistant *B. pertussis* isolates and lack of evidence for such resistance in clinical isolates of the Iranian patients led the authors of the current study to study antibiotic susceptibility of the collected isolates in the country. Susceptibility of the *B. pertussis* isolates to three antibiotics was studied. Relatedness of the strains recovered in this research was also examined.

**Materials and Methods:**

The antibacterial activities of erythromycin, azithromycin, and clarithromycin antibiotics against the recovered isolates of 779 nasopharyngeal swabs were examined using MIC (Minimum Inhibitory Concentration) method. Relationship of the strains was characterized by Pulsed-field Gel Electrophoresis (PFGE).

**Results:**

Among the specimens, 11 cases (1.4%) were culture-positive. Among these isolates, only two isolates had high MIC values for erythromycin and clarithromycin. Pulsed-field gel electrophoresis analysis of the isolates revealed 6 PFGE profiles (A-F) among which three and two isolates had the same patterns in profiles A and B, respectively.

**Conclusions:**

Azithromycin can be a good drug of choice to treat patients infected by *B. pertussis* in Iran. Clonal relationship of the isolates showed that the same *B. pertussis* strains were isolated from different patients in Iran.

## 1. Background

*Bordetella pertussis* causes an important respiratory tract disease known as whooping cough. This illness was considered as one of the major causes of childhood morbidity and mortality worldwide. After introduction of the whole-cell vaccines in 1940s, the rate of pertussis decreased (1). However, the incidence of this contagious disease has been gradually increasing since early 1980s. It seems that there are some compelling explanations for this increase during vaccination programs in the world. It has been suggested that waning immunity along with pathogen adaptation are the main causes for the resurgence of pertussis (2-4). 

Antibiotic treatment is a critical step to prevent the spread of diseases such as pertussis. As pertussis is a highly contagious respiratory disease, monitoring the emergence of *B. pertussis* specially the isolates which showed resistance to antibiotics is very important. Overuse and inappropriate use of antibiotics provide an opportunity for easy spread of these resistant and contagious bacteria in the community.

Among macrolide antibiotics, erythromycin is the first choice of treatment for pertussis infections (5, 6). Since resistance to antimicrobial agents in *B. pertussis* was rare, testing susceptibility to antibiotics in clinical isolates of this bacterium was therefore not deemed necessary (7). Prior to 1994, resistance to erythromycin in *B. pertussis *had not been observed. Since then few reports of resistant isolates of *B. pertussis* to macrolides in some countries such as the United States and Taiwan have been published (8-12). In addition, resistance to quinolones as an alternative drug has also emerged in *B. pertussis* isolates in Japan (13). Mechanisms of resistance in *B. pertussis* are being understood. Recent studies suggest that mutation in the genes related to erythromycin could result in resistance to this macrolide antibiotic (8, 9).

Among various microbial strain-typing methods, Pulsed-field Gel Electrophoresis (PFGE) has been used to establish genetic relationships between the *B. pertussis* strains (14). Some studies have also revealed significant changes in the *B. pertussis* population over time by this useful method (15, 16). Here PFGE was used to study clonality of the isolated strains from Iranian patients.

## 2. Objectives

Previous studies in Iran showed increase of pertussis incidence in the community (17, 18). Recent reports on macrolide-resistant *B. pertussis* isolates and lack of evidence of such resistance in clinical isolates of Iranian patients led the authors to determine antibiotic susceptibility of the collected isolates in the country. Susceptibility of the isolates to erythromycin, azithromycin, and clarithromycin as the drugs of choice to treatment of whooping cough was studied. Finally, relatedness of the isolated clinical strains recovered in this research was examined by PFGE.

## 3. Methods and Materials

### 3.1. Bacterial Isolates

A total of 779 nasopharyngeal swabs were collected from pertussis suspected patients with the age ranging from less than one month to more than ninety years and delivered to the Pertussis Reference Laboratory at the Pasteur Institute of Iran from May 2009 to December 2010. Specimen swabs (Dacron-tipped swabs) were transported from different provinces of Iran to the laboratory on Regan-Lowe transport medium. Specimens were cultured and streaked on fresh Regan-Lowe, and Bordet Gengou mediums (Difco, USA) containing 10% defibrinated horse blood with and without cephalexin (40 µg/mL) (Sigma Chemical Co., USA). After incubation, suspected colonies were confirmed as *B. pertussis* using conventional biochemical tests and specific agglutination reaction with *B. pertussis* antiserum (Difco, USA) (17-20).

### 3.2. Antibiotic Susceptibility Testing

The activities of erythromycin, azithromycin and clarithromycin (Shifa Pharmed Industrial Group, Iran) against *B. pertussis* isolates were examined using agar dilution by the direct suspension method in Muller-Hinton agar (Difco, USA) supplemented with 5% horse blood with incubation temperatures of 35-37°C for a period of 72 hours (21, 22). To determine MIC (Minimum Inhibitory Concentration), the concentrations of antimicrobial agents, range of 0.016 to 512 µg/Ml were prepared. Antibiotic-free plates were inoculated to check for growth and purity. *B. pertussis ATCC* 9797 and *Staphylococcus aureus* *ATCC* 29213 served as the control strains. Strains with macrolide MICs less than or equal to 0.12 μg/ml were considered susceptible (13, 22, 23).

### 3.3. Pulsed field Gel Electrophoresis

*B. pertussis* isolates were characterized by PFGE as described previously (14). The plug slices were incubated overnight at 37°C in 100 µL of buffer solution containing 30 U of XbaI (Fermentase, Vilnius, Lithuania). The pulsed-field program was modified (running times, 19 hours, and pulse times, 5 to 40 seconds, 6 V/cm). The cooling system was set to 14°C. Restricted fragments of chromosomal DNA of the isolates were separated in CHEFF DR-II apparatus (Bio-Rad, USA). *Salmonella braenderup* H9812 was used as molecular weight marker.

## 4. Results

In the current study, out of 779 nasopharyngeal swabs moistened per individual, 11 specimens (1.4%) were culture-positive. Of the total samples examined in the current study, 657 (84.3%) specimens were collected from 10-years-old or younger patients. Ten out of 11 culture-positive strains were isolated from 10-years-old or younger patients. Nine cultures were collected from two-years-old or younger patients.

According to Table 1, MIC determination of the isolates revealed that only two isolates had high MIC values against erythromycin and clarithromycin (MIC ≥ 128 µg/mL). From the 11 strains whose MICs were examined, five and six strains showed MIC ≥ 2 µg/mL against erythromycin and clarithromycin, respectively. Except one isolate with MIC=0.12 µg/mL against azithromycin, all the isolates showed MIC ≤ 0.06 µg/mL against this antibiotic.

**Figure 1.**
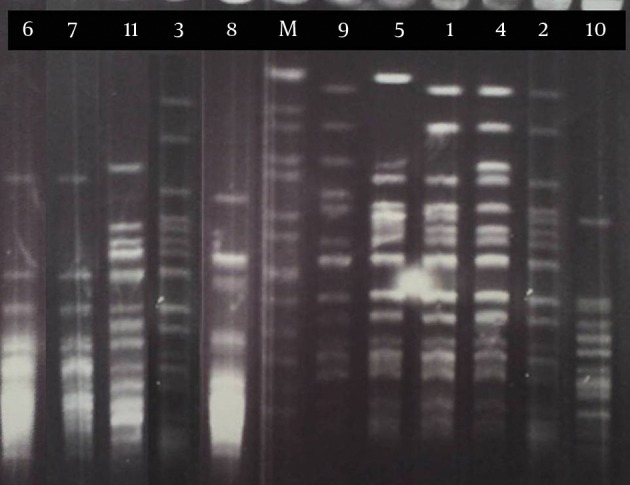
PFGE Pattern of the *B. pertussis *Strains Digested With XbaI Lane M, genomic pattern of *S. braenderup* H9812 as a marker.

Pulsed-field gel electrophoresis analysis of 11 strains of *B. pertussis* in this research revealed six PFGE profiles (A-F) among which three (strains number 1 to 3) and two (strains number 6 and 7) isolates had the same patterns in profiles A1 and B, respectively (Figure 1). These isolates were collected from different provinces of Iran (Table 1). Five closely related isolates (stains number 1, 2, 3, 4 and 5) in profile A with similar susceptibility patterns (except for strain no. 5) were clustered (Table 1). Three of these strains were considered as profile A1 and the others as profiles A2 and A3 according to the minor difference in their banding patterns (Figure 1 and Table 1). In profile B, there were two strains with nearly similar susceptibility patterns isolated from different geographical locations at different dates.

**Table 1.** Antibiotic Susceptibility of Clinical *B. pertussis *Isolates ^a^

Isolates Number	Strains (Age of Patients)	Year (Province of Iran)	PFGE Pattern	MIC		
				Erythromycin	Clarithromycin	Azithromycin
**1**	BpT1 (2 m)	2009 (Tehran)	A1	< 0.06	< 0.06	<0.06
**2**	BpM1 (3 m)	2009 (Mazandaran)	A1	< 0.06	< 0.06	0.125
**3**	BpT2 (3 m)	2009 (Tehran)	A1	< 0.06	< 0.06	< 0.06
**4**	BpE1 (2 y)	2009 (Esfahan)	A2	< 0.06	< 0.06	< 0.06
**5**	BpQ1 (18 y)	2009 (Qom)	A3	< 0.06	2	< 0.06
**6**	BpM2 (3 y)	2009 (Mazandaran)	B	4	8	< 0.06
**7**	BpK1 (5 m)	2010 (Khoozestan)	B	4	32	< 0.06
**8**	BpKO1 (4 y)	2009 (Khorasan)	C	128	> 256	< 0.06
**9**	BpM3 (1 m)	2009 (Mazandaran)	D	< 0.06	0.5	< 0.06
**10**	BpK2 (1.5 m)	2009 (Khorasan)	E	2	<0.06	< 0.06
**11**	BpK3 (26 d)	2009 (Khorasan)	F	128	> 256	< 0.06

^a^ Abbreviations: y, year; m, month; MIC, Minimum Inhibitory Concentration ; PFGE, Pulsed Field Gel Electrophoresis.

Two strains (number 8 and 11) with high level of resistance to erythromycin and clarithromycin (MIC≥128) were isolated from two patients of different ages but living in the same province (Table 1). These two strains had distinct PFGE patterns, suggesting that they were not related. Hopefully, these strains were not among the isolates in profile A that were considered as a predominant profile (with 5 isolates) in this research.

## 5. Discussion

Erythromycin used to be the drug of choice for treatment of pertussis however, because of resistance to this drug, azithromycin has been recently an alternative choice for children (24). In Japan, prophylactic administration of erythromycin caused adverse effects such as digestive organ symptoms, diarrhea, stomachache and abdominal distention in hospital staffs in 2009. It suggested that attention should be paid to erythromycin compliance during a pertussis outbreak (25).

In 2001 in Poland data showed that erythromycin remains the drug of choice for treatment of whooping cough and in case of resistant strains can be replaced by azithromycin. Azithromycin was found the most active antibiotic against *B. pertussis* infection (26). In the U.K in 2010, no evidence of resistance was found in the strains tested against erythromycin, azithromycin and clarithromycin (27). In Taiwan all suspected isolates appeared to be susceptible to erythromycin, azithromycin, clarithromycin and co-trimoxazole (11). In Australia, no significant decrease was observed in the susceptibility to erythromycin among *B. pertussis* strains over 35 years (28).This is the first report on antimicrobial susceptibility of *B. pertussis* isolates in Iran. For evidence of such resistance in clinical isolates of *B. pertussis*, the susceptibility of isolates recovered from clinical specimens to three macrolides was examined.

As expected, the results showed that the rates of resistance to azithromycin were less than those of clarithromycin and erythromycin. Two isolates that indicated high resistance to the two latter antibiotics were susceptible to the former (Table 1). Therefore, similar to the other investigations, the current study data also demonstrated that azithromycin is the most efficient antibiotic against *B. pertussis* isolates (22, 24). Co-trimoxazole is a less active agent against *B. pertussis* strains. However, it can be used in children who cannot tolerate erythromycin or the ones infected by resistant isolates of *B. pertussis* (29).

Since the number of recovered isolates was low, the true rate of resistance could not be found in this research. The current study results suggested that macrolides, especially azithromycin can be a good choice to treat patients infected by *B. pertussis* in Iran. Different mechanisms may confer resistance to erythromycin in the isolates (30). Molecular methods can help to find the reason of resistance even in the absence of the isolates. Resistance to erythromycin is also likely due to a mutation of the erythromycin binding site in 23S rRNA gene (8, 9). In order to find the genetic relationship between the isolates, genomic patterns of the isolates were obtained by PFGE. Even though there are some new typing methods such as MLST (Multi-Locus Sequence Typing) and MLVA (Multiple-Locus Variable-Number Tandem Repeat Analysis) for epidemiologic study, PFGE has been used most widely for typing *B. pertussis* strains in view of its high discriminating power (14, 15, 31). By PFGE, it was shown that an international expansion of *B. pertussis* strains from a similar source resulted in high incidence of pertussis in 1997 in Europe and Taiwan (32).

Pulsed-field gel electrophoresis also found an outbreak and source of infection among *B. pertussis* recovered from Brazilian patients (33). Similarly, in Sweden, Advani. was able to successfully use PFGE as a tool to monitor the bacterial population in vaccine surveillance (14). PFGE also showed expansion of certain PFGE profiles within the *B. pertussis* population from five European countries (34).Based on the current study results, clonal relationship of the isolates showed that the same *B. pertussis* strains were isolated from different patients in Iran; Cluster A, including five strains, was found among the patients from different provinces of the country and may spread more easily than the other PFGE profiles. 

Clonal spread of such strains, transferred from one person to another through the respiratory tract, may cause some important outbreak and should be considered. Fortunately, two highly resistant isolates in this research had unique patterns and had been isolated from different geographical locations. Since the rate of culture-positive strains was low, the obtained data in this research may not give the true incidence of pertussis in Iran. However, these data are the first information on the resistance and spread of *B. pertussis* isolates in different provinces of Iran.

In conclusion, screening for antimicrobial resistance of this respiratory tract infection agent may be warranted especially for strains that showed resistance to macrolides in order to control the spread of these bacteria. Moreover, molecular causes of resistance to antibiotics in the isolates need to be studied further.

## References

[1] Cherry JD (2006). Epidemiology of pertussis.. Pediatr Infect Dis J..

[2] Mooi FR, van Oirschot H, Heuvelman K, van der Heide HG, Gaastra W, Willems RJ (1998). Polymorphism in the Bordetella pertussis virulence factors P.69/pertactin and pertussis toxin in The Netherlands: temporal trends and evidence for vaccine-driven evolution.. Infect Immun..

[3] Mooi FR (2010). Bordetella pertussis and vaccination: the persistence of a genetically monomorphic pathogen.. Infect Genet Evol..

[4] Wood N, McIntyre P (2008). Pertussis: review of epidemiology, diagnosis, management and prevention.. Paediatr Respir Rev..

[5] Bamberger ES, Srugo I (2008). What is new in pertussis?. Eur J Pediatr..

[6] Crowcroft NS, Pebody RG (2006). Recent developments in pertussis.. Lancet..

[7] Lewis K, Saubolle MA, Tenover FC, Rudinsky MF, Barbour SD, Cherry JD (1995). Pertussis caused by an erythromycin-resistant strain of Bordetella pertussis.. Pediatr Infect Dis J..

[8] Bartkus JM, Juni BA, Ehresmann K, Miller CA, Sanden GN, Cassiday PK (2003). Identification of a mutation associated with erythromycin resistance in Bordetella pertussis: implications for surveillance of antimicrobial resistance.. J Clin Microbiol..

[9] Guillot S, Descours G, Gillet Y, Etienne J, Floret D, Guiso N (2012). Macrolide-resistant Bordetella pertussis infection in newborn girl, France.. Emerg Infect Dis..

[10] Korgenski EK, Daly JA (1997). Surveillance and detection of erythromycin resistance in Bordetella pertussis isolates recovered from a pediatric population in the Intermountain West region of the United States.. J Clin Microbiol..

[11] Wilson KE, Cassiday PK, Popovic T, Sanden GN (2002). Bordetella pertussis isolates with a heterogeneous phenotype for erythromycin resistance.. J Clin Microbiol..

[12] Yao SM, Liaw GJ, Chen YY, Yen MH, Chen YH, Mu JJ (2008). Antimicrobial susceptibility testing of Bordetella pertussis in Taiwan prompted by a case of pertussis in a paediatric patient.. J Med Microbiol..

[13] Ohtsuka M, Kikuchi K, Shimizu K, Takahashi N, Ono Y, Sasaki T (2009). Emergence of quinolone-resistant Bordetella pertussis in Japan.. Antimicrob Agents Chemother..

[14] Advani A, Donnelly D, Hallander H (2004). Reference system for characterization of Bordetella pertussis pulsed-field gel electrophoresis profiles.. J Clin Microbiol..

[15] Advani A, Van der Heide HG, Hallander HO, Mooi FR (2009). Analysis of Swedish Bordetella pertussis isolates with three typing methods: characterization of an epidemic lineage.. J Microbiol Methods..

[16] Bisgard KM, Christie CD, Reising SF, Sanden GN, Cassiday PK, Gomersall C (2001). Molecular epidemiology of Bordetella pertussis by pulsed-field gel electrophoresis profile: Cincinnati, 1989-1996.. J Infect Dis..

[17] Nikbin VS, Shahcheraghi F, Lotfi MN, Zahraei SM, Parzadeh M (2013). Comparison of culture and real-time PCR for detection of Bordetella pertussis isolated from patients in Iran.. Iran J Microbiol..

[18] Shahcheraghi F, Nakhost Lotfi M, Parzadeh M, Nikbin VS, Shouraj F, Zahraei S (2012). [Isolation of Bordetella Pertussis and Bordetella Parapertussis from Clinical Specimens at Different Provinces].. J Mazandaran Univ Med Sci..

[19] Koneman EW (2006). Koneman's color Atlas and Textbook of diagnostic microbiology..

[20] McGowan KL, Isenberg HD (2004). Bordetella cultures.. Clinical microbiology procedures handbook..

[21] (2010). Performance standards for antimicrobial susceptibility testing; eighteenth informational supplement.

[22] Hill BC, Baker CN, Tenover FC (2000). A simplified method for testing Bordetella pertussis for resistance to erythromycin and other antimicrobial agents.. J Clin Microbiol..

[23] Hoppe JE, Bryskier A (1998). In vitro susceptibilities of Bordetella pertussis and Bordetella parapertussis to two ketolides (HMR 3004 and HMR 3647), four macrolides (azithromycin, clarithromycin, erythromycin A, and roxithromycin), and two ansamycins (rifampin and rifapentine).. Antimicrob Agents Chemother..

[24] Langley JM, Halperin SA, Boucher FD, Smith B (2004). Azithromycin is as effective as and better tolerated than erythromycin estolate for the treatment of pertussis.. Pediatrics..

[25] Tanaka H, Kaji M, Higuchi K, Shinohara N, Norimatsu M, Kawazoe H (2009). Problems associated with prophylactic use of erythromycin in 1566 staff to prevent hospital infection during the outbreak of pertussis.. J Clin Pharm Ther..

[26] Chodorowska M, Kuklinska D, Tyski S (2001). [Susceptibility to macrolide antibiotics of Bordetella pertussis and Bordetella parapertussis strains isolated from whooping cough patients in 1968 and in 1997-99].. Med Dosw Mikrobiol..

[27] Fry NK, Duncan J, Vaghji L, George RC, Harrison TG (2010). Antimicrobial susceptibility testing of historical and recent clinical isolates of Bordetella pertussis in the United Kingdom using the Etest method.. Eur J Clin Microbiol Infect Dis..

[28] Sintchenko V, Brown M, Gilbert GL (2007). Is Bordetella pertussis susceptibility to erythromycin changing? MIC trends among Australian isolates 1971-2006.. J Antimicrob Chemother..

[29] Hoppe JE, Halm U, Hagedorn HJ, Kraminer-Hagedorn A (1989). Comparison of erythromycin ethylsuccinate and co-trimoxazole for treatment of pertussis.. Infection..

[30] Weisblum B (1995). Erythromycin resistance by ribosome modification.. Antimicrob Agents Chemother..

[31] Mooi FR, Hallander H, Wirsing von Konig CH, Hoet B, Guiso N (2000). Epidemiological typing of Bordetella pertussis isolates: recommendations for a standard methodology.. Eur J Clin Microbiol Infect Dis..

[32] Lin YC, Yao SM, Yan JJ, Chen YY, Hsiao MJ, Chou CY (2006). Molecular epidemiology of Bordetella pertussis in Taiwan, 1993-2004: suggests one possible explanation for the outbreak of pertussis in 1997.. Microbes Infect..

[33] Goncalves CR, Vaz TM, Medeiros MI, Castro MT, Rocha MM, Melles CE (2007). Phenotypical and genotypical characterization of Bordetella pertussis strains isolated in Sao Paulo, Brazil, 1988-2002.. Rev Inst Med Trop Sao Paulo..

[34] Hallander H, Advani A, Riffelmann M, von Konig CH, Caro V, Guiso N (2007). Bordetella pertussis strains circulating in Europe in 1999 to 2004 as determined by pulsed-field gel electrophoresis.. J Clin Microbiol..

